# Diabetic Mastopathy: A Monocentric Study to Explore This Uncommon Breast Disease

**DOI:** 10.3390/diagnostics14232749

**Published:** 2024-12-06

**Authors:** Luciano Mariano, Luca Nicosia, Sofia Scolari, Sara Pasi, Sofia Netti, Giovanni Mazzarol, Antuono Latronico, Enrico Cassano

**Affiliations:** 1 Breast Imaging Division, Radiology Department, IEO European Institute of Oncology IRCCS, 20141 Milan, Italy; luca.nicosia@ieo.it (L.N.); antuono.latronico@ieo.it (A.L.); enrico.cassano@ieo.it (E.C.); 2 Postgraduation School in Radiodiagnostics, Università degli Studi di Milano, Via Festa del Perdono 7, 20122 Milan, Italy; sofia.scolari@unimi.it (S.S.); sara.pasi@unimi.it (S.P.); 3 Molecular and Pharmaco-Epidemiology Unit, Department of Experimental Oncology, IEO European Institute of Oncology IRCCS, 20141 Milan, Italy; sofia.netti@ieo.it; 4 Division of Pathology, IEO European Institute of Oncology IRCCS, 20141 Milan, Italy; giovanni.mazzarol@ieo.it

**Keywords:** diabetic mastopathy, diabetes mellitus, breast cancer, breast imaging

## Abstract

Background: Diabetic Mastopathy (DMP) is an uncommon benign fibro-inflammatory condition that occurs in women with long-standing diabetes mellitus (DM), particularly type 1. It often mimics breast cancer (BC) in clinical and imaging presentations, leading to diagnostic challenges. Methods: A retrospective monocentric study was conducted, analyzing clinical, radiologic, and pathological data from 28 women diagnosed with DMP over 10 years at the European Institute of Oncology. Data on DM type, age at DMP diagnosis, associated autoimmune conditions, imaging features, and surgical outcomes were collected and compared with the existing literature. Results: The majority (82%) of the patients had type 1 DM, with most diagnosed with DMP before age 40. Common complications included retinopathy (46%) and neuropathy (35%). Imaging often suggested malignancy, necessitating core needle biopsies for diagnosis. Surgical intervention occurred in 55% of cases, with a recurrence rate of 32%. One case of BC was observed. Conclusions: DMP remains challenging due to its resemblance to BC. Conservative management is typical, but the recurrence rate post-surgery highlights the importance of ongoing monitoring. Although DMP does not significantly increase BC risk, caution is advised, especially for immunocompromised patients. Further studies are needed to comprehensively understand DMP’s relationship with BC.

## 1. Introduction

Diabetic Mastopathy (DMP) is a rare benign fibro-inflammatory condition that primarily affects women with long-standing diabetes mellitus (DM), particularly type 1. Despite its benign nature, DMP often mimics breast cancer (BC) both clinically and radiologically, posing a significant diagnostic challenge for clinicians [1,2].

A painless, irregular, and palpable mass (or multiple masses) in one or both breasts, often recurring, constitutes the typical clinical presentation [3,4]. The imaging features may also resemble BC, such as spiculated margins and heterogeneous echogenicity on ultrasound (US) [5], often necessitating a multidisciplinary approach involving clinical evaluation, imaging studies, and pathological correlation to confirm a definitive diagnosis and exclude malignancy [6,7]. 

Histopathologically, DMP is characterized by sclerosing lymphocytic lobulitis. Although this pattern is crucial for accurate DMP diagnosis in DM patients, it is not exclusive to this metabolic disease, as similar features may be found in other autoimmune disorders [8,9]. Given its potential to be misdiagnosed as BC, DMP often leads to unnecessary surgical interventions despite its non-malignant nature.

Although several studies on DMP have been published, most have focused on single cases without systematically analyzing the clinical implications of DMP. Moreover, there is a lack of consolidated evidence clarifying the relationship between DMP and the risk of developing BC, particularly in immunocompromised patients or those with autoimmune comorbidities. 

This study aimed to address this gap by examining the clinical, radiologic, and pathological data of a monocentric cohort of patients diagnosed with DMP at our institute and comparing them with the existing literature. Through this comparative investigation, we aimed to contribute to a better understanding of the variability and peculiarities in the presentation and management of DMP, thus providing a significant contribution to the current body of knowledge in this field and assisting healthcare professionals in the practical direction of these patients.

## 2. Materials and Methods

This retrospective monocentric study, based on a non-consecutive sampling of women, gathered data from patients diagnosed with DMP who attended the Breast Imaging Division at the European Institute of Oncology of Milan over the past 10 years (December 2013–December 2023). This study included 28 women with histologically confirmed DMP.

The inclusion criteria comprised a DM diagnosis according to the American Diabetes Association guidelines [10], a breast core biopsy with a histological pattern of lymphocytic sclerosing lobulitis, and informed consent. 

Clinical data were collected from the patients’ medical records, imaging findings from RIS/PACS systems, and histopathological data obtained via core needle biopsy or excisional biopsy. All information was recorded in a dedicated spreadsheet.

Clinical categories were analyzed, including sex, ages at DM and DMP diagnoses, DM type, presence of concomitant autoimmune diseases, and any associated chronic DM complications. 

Features obtained through different imaging techniques, selected based on clinical indications (Digital Mammography (MG), US, and Magnetic Resonance Imaging (MRI)), were evaluated for parameters such as breast density, lesion site, size, lesion type, margins, and BI-RADS score [11]. Additionally, any surgical interventions were assessed, specifying the type of procedure (mastectomy, resection, or lumpectomy), possible disease recurrence, and the development of BC, with the latter evaluated based on histotype and Grading (low, G1; intermediate, G2; high, G3) [12].

Data collection and analysis were conducted using a descriptive statistical approach. The results are presented in percentage form to provide a comprehensive and accurate overview of the information obtained during this study. Continuous variables are summarized as medians with interquartile ranges (IQRs), while categorical variables are presented with absolute and relative frequencies. Inferential statistical techniques were not used to assess associations or significant differences due to the limited sample size (28 patients), which reduced statistical power and increased the risk of non-representative or non-significant results in inferential analyses.

## 3. Results

Our DMP study sample comprised exclusively female patients (*n* = 28) with long histories of DM [median time between DM and DMP: 15 years (IQR: 11, 22)]. 

Most of the patients (82%) had DM1; one woman had a medical history that included DM2 and a late (62 years old) DMP diagnosis. The majority, 23 (82%), were diagnosed with DMP before 40 years old. In addition, 73% (19/26) of the cohort presented at least one additional DM-related complication, most commonly retinopathy (46%), followed by neuropathy (35%) and nephropathy (23%). Concomitant autoimmune conditions were identified in 11/26 patients (42%), with thyroid disorders being the most prevalent (9/26). 

Imaging modalities frequently suggested malignancy, with poorly defined masses and extensive fibrosis seen on both ultrasound and MRI. 

Regarding the surgical outcomes, 55% (15/27) of the women were treated using surgical approaches. Among these, 80% opted for surgical resection, 13% experienced a mastectomy, and only 6% underwent lumpectomy. The remaining 12 patients adhered to regular clinical and instrumental monitoring. In total, 32% of the women experienced a DMP recurrence after surgery, primarily within the first year following the procedure. 

The main clinical data that were collected are presented in the following table (Table 1).

## 4. Discussion

DMP is an uncommon benign breast condition that occurs in longstanding DM patients. Its etiology is not fully understood, and its pathogenesis is probably immune-mediated. It features painless lumps and imaging features that mimic BC [1,2]. Sclerosing lymphocytic lobulitis, often used synonymously, can confuse physicians [8]. While key for accurate DMP diagnosis in DM patients, it is not exclusive to this metabolic disorder and may also appear in women with other autoimmune diseases [9].

First described by Soler and Khardori in 1984 [13] and later by Byrd in 1987 [14] as painless fibrous breast masses with perivascular lymphocytic infiltrates in women with DM1, Tomaszewsky coined the term DMP five years later, identifying epithelioid fibroblast cells in over 75% of pathological stains from DMP patients [15].

DMP accounts for less than 1% of all benign breast lesions, primarily affecting premenopausal women in their 30s and 40s with longstanding DM1 [2,13,16,17]. Onset typically occurs 15–20 years after DM diagnosis [18], with around 70% of patients also presenting associated microvascular complications, such as diabetic nephropathy, neuropathy, or retinopathy [17,19]. Rare cases have been reported in DM2 patients [9] and in men with gynecomastia-like presentations [20,21]. 

**Figure 1:**
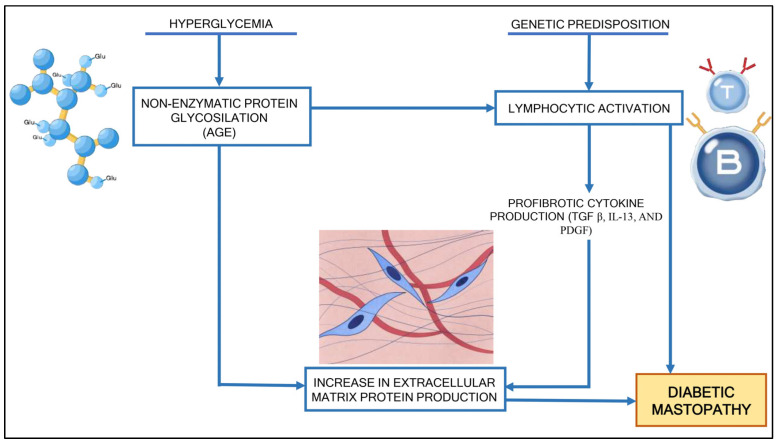
Pathogenetic DMP theory. Hyperglycemia promotes non-enzymatic protein glycosylation with glycosylation end-product (AGE) formation. In genetically predisposed individuals, AGEs selectively bind to a specific receptor (RAGE) on inflammatory cells, triggering cytokine release and excessive extracellular matrix production. AGEs can also bind extracellular matrix proteins, which become more resistant to proteolytic action and increase their accumulation.

The relationship between DM and the development of DMP is not fully understood, but key hypotheses suggest prolonged hyperglycemia leads to non-enzymatic protein glycation and neoantigen formation, triggering an autoimmune response [16,17,22]. Evidence supporting these hypotheses includes chronic inflammation with dense periductal, perilobular, and perivascular lymphocytic infiltrates seen in DMP lesions [23,24,25] (pp. 53–56, [24]); expression of HLA-DR3, 4, or 5 phenotypes in affected lobular units [26]; and associations with autoimmune diseases like Sjogren’s syndrome, parotitis, and thyroiditis [9,27]. Glycated proteins form cross-links in the breast stroma, inducing a humoral response and B-lymphocyte activation, followed by inflammation, lymphokine secretion, fibroblast activation, and increased collagen deposition [15,16] (Figure 1). An alternative theory proposes cross-reactivity between anti-insulin autoantibodies and duct cells due to insulin supplementation [22,23], though cases of DMP in diabetic women that do not use insulin, including in DM2, challenge this idea.

Patients with DMP present painless, firm, and poorly demarcated masses that mimic BC but typically lack axillary lymphadenopathy [3]. The masses may be solitary or multiple, affecting one or both breasts, with a tendency to recur and grow within the first year post-diagnosis [15].

**Figure 2:**
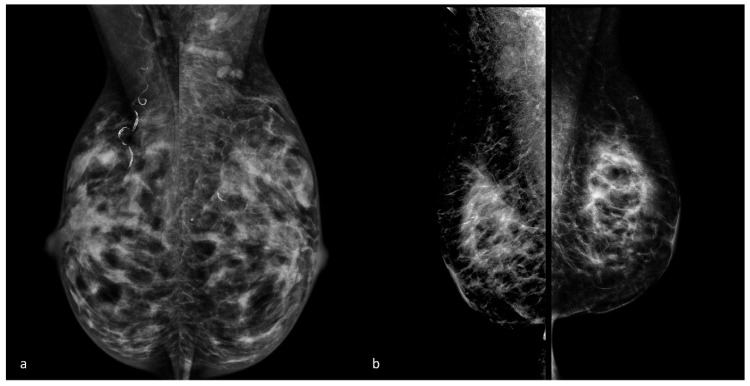
(**a**) A 47-year-old female during insulin therapy with no breast symptoms, a negative BC family history, and a DMP diagnosis following core needle biopsy. Medio-lateral oblique 2D mammograms show extremely dense breasts without suspicious radiologic findings. Vascular calcifications are observed in the upper quadrants of the right breast. Benign vascular calcifications may be frequently observed in DM women, although they are not explicitly associated with DMP. (**b**) A 39-year-old female during insulin therapy with painless and poorly demarcated bilateral masses on clinical examination and a DMP diagnosis following core needle biopsy. Medio-lateral oblique 2D mammograms show multiple bilateral glandular thickenings with skin retraction in the lower quadrants, mimicking BC.

**Figure 3:**
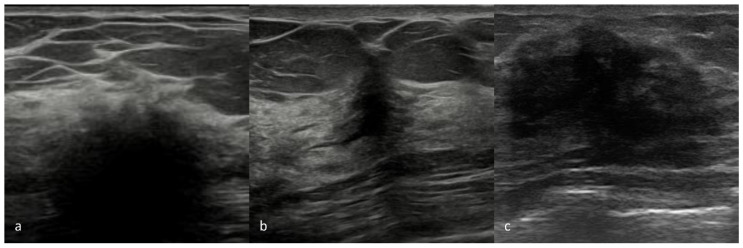
B-mode ultrasound images of a 34-year-old female with DM1, Hashimoto’s disease, and a DMP diagnosis following core needle biopsy show inhomogeneous hypoechogenic areas with irregular margins (**a**) and irregular hypoechogenic areas with indistinct margins, a non-parallel orientation, and posterior acoustic shadowing (**b**), indicating DMP lesions. (**c**) Inhomogeneous hypoechogenic areas with irregular margins of a 39-year-old woman with DM1 and an invasive ductal carcinoma diagnosis who underwent core needle biopsy.

**Figure 4:**
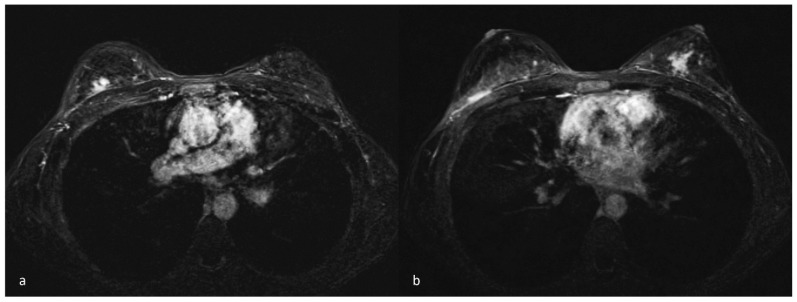
MRI subtraction images of a 38-year-old woman with a DMP diagnosis following a core needle biopsy show two inhomogeneous mass-like enhancements with a persistent kinetic pattern, irregular shapes, and spiculated margins in the upper outer quadrant of the right breast (**a**) and in the retroareolar areas of the left breast (**b**).

**Figure 5:**
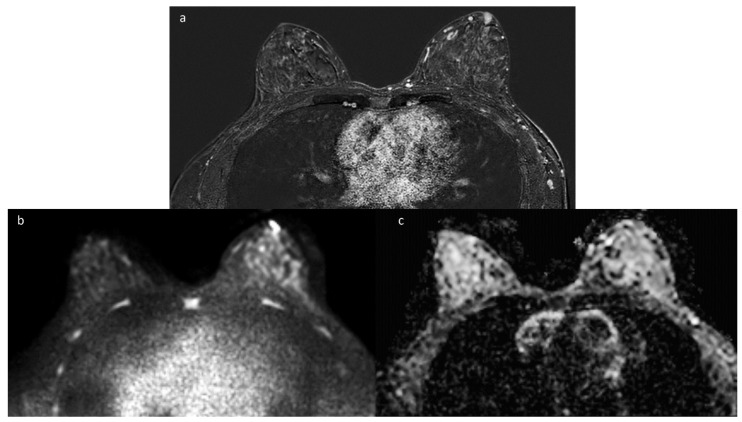
MRI subtraction image (**a**) of a 35-year-old woman with a DMP diagnosis following a core needle biopsy shows a heterogeneous mass-like enhancement with an oval shape in the superficial retroareolar areas of the left breast. Hyperintensity on DWI sequences (**b**) and signal restriction in an ADC map (**c**) mimic BC.

**Figure 6:**
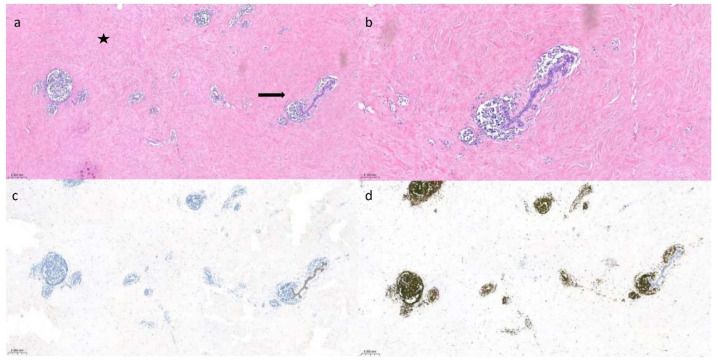
Hematoxylin and eosin staining (**a**) of a histological section of DMP (5×) shows perivascular and periductal lymphoid infiltrates (arrow) in the expanded collagenous stroma (star). (**b**) Higher magnification (10×) shows mature lymphoid infiltrates in and around a duct lobular unit. (**c**) Immunohistochemistry for Keratin (5×) highlights the epithelial glandular component. (**d**) Leukocyte Common Antigen (LCA) immunohistochemistry (5×) depicts perivascular and periductal mature lymphoid infiltrates.

Due to clinical suspicion of an underlying malignancy, imaging studies, such as MG and US, are essential for initial assessment, though MG findings often lack specificity and accuracy, showing poorly defined solid lesions, asymmetrical densities, or architectural distortions [7] (Figure 2). US may reveal indistinct hypoechogenic areas with distinctive acoustic shadowing and no vascularity, likely due to fibrosis [28,29] (Figure 3). MRI can aid in distinguishing DMP from BC, with DMP lesions exhibiting a gradually persistent enhancing kinetic pattern, represented by a time–intensity curve typical of a benign lesion [17,30]. However, lesions with significant lymphocytic infiltration can sometimes create hyperintense signals on DWI sequences, complicating differentiation from malignancy [31] (Figure 4 and Figure 5). Core needle biopsy is often necessary for a final diagnosis, as fine-needle aspiration is typically inconclusive due to low cellularity. Nevertheless, dense fibrosis can pose challenges in obtaining sufficient tissue for an accurate diagnosis, even by needle biopsy, necessitating an excisional biopsy [14,29]. Histologically, DMP lesions feature dense fibrosis and chronic inflammation. Stroma changes include intense fibroblast and myofibroblast proliferation with abnormal dense collagen deposition (keloid-like). Atrophic ductules and lobules with thick basal laminae (sclerotic lobulitis) are often associated with DMP; sometimes, fibroblasts present enlarged pleomorphic nuclei and abundant cytoplasm, resembling epithelioid cells [15]. Dense lymphocytic infiltrates, primarily B cells, are always detected around lobules, ducts, and vessels, creating a “sclerosing lymphocytic lobulitis” pattern [32] (Figure 6).

Various proposed diagnostic criteria for DMP have emerged over the years. Camuto et al. outlined essential points for correct DMP identification [16] (Table 2). In addition, a summary of Tomaszewski’s classification highlights four key pathological features of DMP [15] (Table 3).

**Table 2:** Camuto’s diagnostic criteria.

Diagnostic Criteria for Diabetic Mastopathy (Camuto et al. [16])
1. Premenopausal women with long-standing type 1 DM, often with associated vascular complications.
2. Palpable breast lesion is firm, painless, and clinically raises suspicions of breast cancer.
3. Increased density on mammography without a localized mass, and ultrasound similarly fails to detect a solid or cystic mass.
4. Areas of fibrosis linked with perivascular lymphocytic infiltration via surgical or thick-needle biopsy.

**Table 3:** Tomaszweski’s pathological features.

Pathological Features of Diabetic Mastopathy (Tomaszweski et al. [15])
1. Lymphocytic lobulitis and ductitis with glandular atrophy
2. Lymphocytic/mononuclear perivascular inflammation, predominantly B cells
3. Dense, keloid-like fibrosis
4. Epithelioid-like fibroblasts

DMP management generally involves a combination of conservative and surgical approaches. Since most DMP lesions are benign, once a diagnosis has been made, conservative assessment with age-appropriate routine MG and clinical evaluation of palpable abnormalities is recommended [6,33]. Although spontaneous regression of DMP may occur, patients remain at risk of BC, and it is not uncommon for new masses to be found during follow-up imaging examinations. Thus, any newly identified mass should be carefully evaluated, with additional biopsies considered if needed (pp. 55–56, [24]). 

However, in special cases such as unresolved malignancy concerns, continuously growing masses, cosmetic concerns, or patient anxiety, a surgical approach may be necessary to rule out malignancy and alleviate any associated symptoms [34]. 

Surgical approaches should be planned to achieve the best quality of life and optimal aesthetic outcomes. Surgical excision with an adequate breast tissue margin remains the primary treatment option; however, a high recurrence rate (30%) of DMP masses has been reported [4,28], most often near the original site [35].

Our findings are consistent with previously published studies, affirming the association of DMP with longstanding DM1 [82% of patients, with the median time between DMP and DM diagnoses being 15 years (IQR: (11, 22))], onset in young adulthood [23/28 (82%) with DMP diagnosis before 40 y.o.], and its autoimmune pathogenesis. The frequent occurrence of autoimmune diseases among our cohort (42%), particularly thyroid disorders (9/26), supports the immunological hypothesis. This observation suggests that DMP could potentially serve as the initial clinical manifestation on an autoimmune basis in some of these patients, especially considering the young age at which it is diagnosed.

Despite the benign nature of DMP, the surgical approach was also common in our investigation. In total, 55% (15/27) of the patients underwent surgery, primarily due to patient anxiety and the difficulty in excluding malignancy based solely on imaging. The recurrence rates are noteworthy, with 32% of the women experiencing a DMP recurrence; notably, 57% encountered recurrence after surgical resection. These findings underscore the importance of considering the recurrence risk and exploring therapeutic alternatives to ensure optimal patient outcomes and address the challenges associated with DMP management.

### Breast Cancer Correlation

To date, no evidence suggests that DMP predisposes individuals to BC (pp. 55–56, [24]). A retrospective multicentric study by Kudva et al. involving 68 patients over a 10-year period reported a BC risk comparable with that of the general population [34]. Additionally, population-based investigations showed no increased risk of BC among women with type 1 or 2 DM [36].

A single case of BC arising from a diabetic fibrous mass in a DMP patient who was a renal transplant recipient was reported [37]. According to transplant tumor registries, the BC incidence in renal transplant recipients is lower than in the general population [38,39,40]. However, these recipients exhibit higher mortality rates when diagnosed with stage III or IV BC, suggesting that immunosuppression may enhance the biological aggressiveness of malignancies [38]. 

Our findings align with the existing literature. Among the 28 women studied, 3 were diagnosed with invasive ductal carcinoma (grades G2 to G3). None had major risk factors typically associated with BC, such as genetic mutations, family history, or prior chest irradiation. Nevertheless, two of these cases had previous episodes of infective endocarditis, emphasizing the immunosuppressive effects correlated with DM and susceptibility to develop infections. According to a study featured in the esteemed Diabetes Care journal in January 2018, individuals with DM1 face a fourfold higher likelihood of hospitalization due to infections, while those with DM2 have twice the probability compared to individuals without DM [41]. Another extensive retrospective investigation involving the cross-referencing of general practitioner records with the hospital clinical files of 102.493 British adults aged 40 to 89 that had been diagnosed with DM since 2008 (*n* = 5.863 for DM1 and *n* = 96.630 for DM2) unveiled that around 6% of hospitalizations were connected to infectious diseases. Moreover, 12% of deaths attributed to infections were found to be linked to DM [42].

The increased susceptibility to infections in individuals with DM is ascribed to widespread hyperglycemia, leading to immune system dysfunction, inhibition of antioxidant mechanisms, and compromised humoral immunity [43]. More extensive studies with longer follow-ups are needed to investigate the relationship between DMP and BC. In light of this, one should be more cautious when managing DMP patients, particularly if they are immunosuppressed.

Nevertheless, some considerations need to be addressed. The relatively small sample size of 28 patients in this monocentric study represents a limitation that could impact the generalizability of the findings. Indeed, although the data provide valuable insights into DMP patients, the restricted population may not adequately capture the broader spectrum of clinical and pathological variations across different demographics or healthcare settings. Moreover, this study’s retrospective design could have introduced selection bias, as it depended on the availability and quality of past data. Addressing these limitations in future studies by including larger multicentric cohorts and prospective methodologies would enhance the robustness and applicability of the results. 

## 5. Conclusions

Our investigation into DMP contributes to our understanding of this uncommon DM complication. Through a comparative analysis of data collected from 28 patients over 10 years, we reaffirm the complexity of diagnosing DMP, emphasizing its similarity to BC. Conservative management remains standard practice, but our observation of a notable recurrence rate after surgical intervention highlights the need for long-term monitoring. Despite a lack of evidence suggesting a predisposition for DMP-to-BC development, we recommend caution, especially in immunocompromised patients, and propose further long-term studies to delve deeper into this intricate relationship.

## Figures and Tables

**Table 1:** The main clinical data of the study patients.

	*n* = 28
**Diabetes Mellitus**	
Type 1 Diabetes Mellitus	26 (96%)
Type 2 Diabetes Mellitus	1 (3.7%)
Missing	1
**Age at DMP diagnosis**	34 (31,38)
**DMP diagnosis before/after 40 y.o.**	
Before 40 y.o.	23 (82%)
After 40 y.o.	5 (18%)
**Years between DMP and DM diagnoses**	15 (11,22)
Missing	2
**Complications**	total: 19/26
Nephropathy	6/26 (23%)
Neuropathy	9/26 (35%)
Retinopathy	12/26 (46%)
No	7/26 (27%)
Missing	2
**Associated autoimmune diseases**	total: 11/26
Thyroiditis	9/26 (35%)
Iridocyclitis	1/26 (3.8%)
Gastritis	1/26 (3.8%)
Psoriasis	1/26 (3.8%)
Vasculopathy (Systemic Lupus Erythematosus)	1/26 (3.8%)
No	15/26 (58%)
Missing	2
**Surgery after DMP**	
Lumpectomy	1 (3.7%)
Mastectomy	2 (7.4%)
Resection	12 (44%)
No	12 (44%)
Missing	1
**Recurrence of DMP**	
No	15 (68%)
Yes	7 (32%)
Missing	6
**Breast Cancer after DMP**	
Yes (Invasive Ductal Carcinoma, Grade 2)	1 (3.8%)
Yes (Invasive Ductal Carcinoma, Grade 3)	2 (7.7%)
No	23 (88.5%)
Missing	2
1 *n* (%); Median (IQR)	

## Data Availability

The data presented in this study are available on request from the corresponding author. The data are not publicly available due to privacy concerns, in accordance with the GDPR.
